# Promoting Nutrition and Food Sustainability Knowledge in Apprentice Chefs: An Intervention Study at The School of Italian Culinary Arts—ALMA

**DOI:** 10.3390/nu16040537

**Published:** 2024-02-15

**Authors:** Cinzia Franchini, Beatrice Biasini, Francesca Giopp, Alice Rosi, Francesca Scazzina

**Affiliations:** 1 Human Nutrition Unit, Department of Food and Drug, University of Parma, 43121 Parma, Italy; cinzia.franchini@unipr.it (C.F.); beatrice.biasini@unipr.it (B.B.); francesca.scazzina@unipr.it (F.S.); 2 ALMA S.r.l., The School of Italian Culinary Arts, 43052 Parma, Italy; nutrizione@scuolacucina.it

**Keywords:** nutrition, food knowledge, nutrition education, sustainable diet, questionnaire, culinary students

## Abstract

Chefs’ decisions can greatly improve the quality of food provided and positively guide diners’ choices. Culinary students’ knowledge of healthy and sustainable nutrition is still scarcely investigated and is limited to the nutritional aspect of the diet, without considering food sustainability or the environmental impact of foods. This study aims to determine the effectiveness of an educational program designed for apprentice chefs. Two questionnaires were administered twice to each student who followed dedicated lectures about nutrition and food sustainability and to other students enrolled as the control group. A total of 264 and 252 apprentice chefs of The School of Italian Culinary Arts—ALMA were enrolled in the control and intervention groups, respectively. At baseline, both groups showed a good level of nutrition knowledge, whereas food sustainability knowledge was lower in all students, regardless of the group. This educational intervention proved to be effective in improving knowledge about nutrition and the environmental impact of food production and consumption. However, a small but significant improvement in nutritional knowledge was also found over time in the control group. Finally, a food sustainability knowledge questionnaire was developed and validated for this study, providing interesting results to be treated as a guide for future developments.

## 1. Introduction

The sustainability of people’s diets and, more in general, of food systems is increasingly a priority to ensure people’s health and preserve the planet’s ecosystems for the next generations. On the contrary, unhealthy diets characterized by high levels of salt and poor consumption of plant-based products, such as whole grains, fruit, vegetables, legumes, nuts, and seeds, represent one of the top risk factors globally for mortality and morbidity [1], negatively influencing the economic balance by increasing the costs of public health. In parallel, the agri-food system is responsible for negative externalities contributing to land degradation, water use and pollution, biodiversity loss, and climate change [2]. Per unit mass (g), the production of animal-based foods has a significantly higher detrimental impact on the environment compared to plant-based products [3], for which a positive effect on non-communicable disease (NCD) incidence reduction is also recognized [4].

Given this, promoting a nutritional transition must necessarily include a change in individuals’ eating habits, and it should be based on several strategies, including improving the knowledge and awareness of multiple food system stakeholders to create a single sustainability vision [5,6]. Due to the numerosity of facilities that provide food to communities, food services can reach a huge number of people, covering a crucial role in this transition process toward more resilient food systems [7,8]. Raw material selection, menu development, and customer outreach contribute to conscious cuisine that supports and facilitates healthy and sustainable food selections. Thus, chefs’ decisions can greatly improve the quality of the food provided and positively influence customers’ dietary choices [8,9]. In addition, the chef, as a figure, has increasingly become a reference not only for food preparation but also for aspects of human nutrition [10,11], extending its impact beyond the kitchen and increasing its ability to positively influence people’s beliefs, business interests, and government actions [10]. This reinforces the key role of chefs as relevant stakeholders in promoting healthy and sustainable foods as a possible solution to address current nutritional and climate emergencies. Therefore, the training of future chefs should cover the practical aspects of restaurant management, such as food procurement and menu preparation, as well as improving their knowledge and dissemination skills [9].

In this connection, culinary students consider nutrition and sustainability as important aspects of dish development, recognizing the responsibility of their choices in influencing future food systems [12,13]. Although several culinary schools and organizations are now including nutrition and sustainability in their training programs [14,15,16,17], culinary students’ knowledge of healthy and sustainable nutrition is still scarcely investigated and is limited to the nutritional aspect of the diet, without considering food sustainability or the environmental impact of foods [18,19].

In this context, this intervention study aims to determine the effectiveness of an educational program in a sample of apprentice chefs through the assessment of nutrition and food sustainability knowledge before and after the teaching curriculum, which included lectures and a menu preparation activity. In addition, canteen users’ satisfaction with the menus was evaluated to determine the acceptability of the prepared meals.

## 2. Materials and Methods

### 2.1. Setting and Study Design

**Figure 1:**
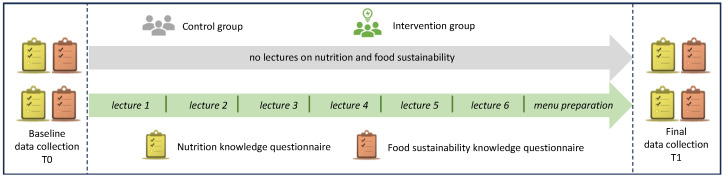
Diagram of the study design.

This intervention study was conducted at The School of Italian Culinary Arts—ALMA, based in Colorno (Parma, Italy), recognized as the premier education and training center for the Italian restaurant and hospitality industries at the international level, between April 2021 and October 2022. During this period, all students (18–39 years) registered for the Cooking Techniques, Basic Pastry Techniques, or Advanced Course of Italian Cuisine course at ALMA were invited to participate in the study at the beginning of the course by the nutrition teacher at the school involved in the research project. To obtain a representative sample of ALMA students (*n* = 966, data referring to year 2019), a sample size of at least 275 students was set, taking into account a confidence level of 95% and confidence interval of 5%. The education intervention was carried out in the Advanced Course of Italian Cuisine as part of the regular teaching program by a teacher in Human Nutrition affiliated with ALMA, and the students taking the course were recruited as the intervention group. On the contrary, the curricula of the Cooking Techniques and Basic Pastry Techniques courses focus on basic knowledge and skills related to the fundamentals of cooking and pastry, respectively, without including lectures on nutrition and food sustainability; therefore, the students who attended them were enrolled as the control group. The flow chart of the study design is presented in Figure 1.

The intervention consisted of 24 h of classroom lectures. During the lessons, instructions about designing and preparing nutritionally balanced and eco-friendly menus were provided to students, such as the energy and macronutrient contents that a balanced meal should have and the environmental impacts of different types of foods. In addition, students were provided with the Food Composition Database for Epidemiological Studies in Italy [20] and the environmental pyramids of carbon (g CO_2_ eq) and water (L) footprints designed by the Barilla Foundation [21].

Briefly, the students of the intervention group were divided into different sub-groups of 4 or 5 subjects who were asked to put into practice the knowledge and skills they have acquired by developing healthy and sustainable menus for a business lunch intended for all ALMA staff and students. Each meal consisted of a small appetizer, a main course, and a fruit-based dessert with reduced sugar and saturated fatty acid (SFA) contents. A meat-, fish-, or plant-based (i.e., vegetarian or vegan) menu was assigned to each sub-group. Only those menus considered to be technically and nutritionally adequate, and with a proper presentation of the dishes, received approval by the teaching staff. In addition, the approved menu recipes were analyzed by researchers at the University of Parma in terms of their nutritional composition, energy and macronutrient contents, and environmental impact, by estimating the carbon (g CO_2_ eq) and water (L) footprint. The Food Composition Database for Epidemiological Studies in Italy [20] and a dataset collecting greenhouse gas emissions and water use linked to the production and the subsequent steps of the supply chains of food commodities, up to distribution [22], respectively, were used. Finally, the menus were prepared and served at the school canteen through lunch boxes. This service mode was preferred over a buffet to reduce the risk of infection due to the COVID-19 health emergency. In the lunchroom, each menu was described considering the number of portions of fruit and vegetables provided compared to the national dietary guidelines, the environmental impacts expressed as kilometers traveled by car for the carbon footprint, and number of water bottles for the water footprint. In addition, the most environmentally sustainable menu with the best nutritional composition among the different proposals (i.e., meat-, fish-, and plant-based options) was labeled with a logo. Lastly, after the consumption of the lunch menus, the canteen users (i.e., students, school staff, or guests) used a quick response (QR) code to access a short questionnaire aimed at investigating the most selected menu category, their enjoyment of the chosen menu, and whether they consumed the entire meal, as well as their perception about the adequacy of the served portion size and their satisfaction with the selected menu assessed on a 5-point scale.

To evaluate changes in students’ knowledge about nutrition and food sustainability, two online questionnaires were administered twice to each participant, at the beginning (1st—baseline—data collection, T0) and the end of the intervention (2nd—final—data collection, T1). In addition, personal information such as gender, age, and educational background was collected for each participant. Before filling out the questionnaire, all participants gave their approval by signing the informed consent form. The study was conducted in accordance with the Declaration of Helsinki, and the study protocol was approved by the Ethical Committee of the Area Vasta Emilia Nord (Prot. n. 41959/2021). Moreover, subjects were asked to identify themselves with an alphanumeric code, the same for both data collections, to guarantee data privacy.

### 2.2. Nutrition and Food Sustainability Knowledge Assessment

The participants’ nutrition knowledge was assessed through a questionnaire already validated for Italian university students [23] of a similar age group as the current study population. The Nutrition Knowledge questionnaire (90 items) includes five constructs addressing the following main topics: experts’ recommendations (6 items), nutritional content of food (49 items), health aspects of food and diet (10 items), relationship between diet and diseases (19 items), and proper food choices (6 items). According to the section, questions were multiple-choice, providing four to six possible answer options, or dichotomous, offering two possible answer options (i.e., High content or Low content, Yes or No). One point was assigned to each correct answer; therefore, the level of nutrition knowledge was assessed on a score from 0 to 90 points. Along with the evaluation of nutrition knowledge, understanding of the main aspects related to sustainable diets and the environmental impact of foods was investigated with an original questionnaire specifically designed for the study. The final questionnaire, composed of 23 questions, was divided into two constructs. Construct number 1 (Basic notions about food sustainability) was composed of 9 multiple-choice questions related to the concept of sustainable diet, the Double Pyramid model, and indicators of environmental sustainability (i.e., carbon, water, and ecological footprint). Among the 9 questions, 5 presented 4 options with 1 correct answer and 1 ‘I do not know’, while the remaining 4 questions included 3 options: ‘True’, ‘False’, or ‘I do not Know’. Construct number 2 (Environmental impact of food) consisted of 14 multiple-choice items on the environmental sustainability of different food commodities, the connection between human and planetary health, and food waste. Four questions offered 4 possible options with 1 correct answer and an ‘I do not know’. The other 10 questions provided 3 options: 5 questions had ‘High impact’, ‘Low impact’, or ‘I do not know’; 4 questions had ‘I agree’, ‘I disagree’, or ‘I do not know’; and 3 questions had ‘True’, ‘False’, or ‘I do not know’ as possible answers. Like for the nutrition knowledge questionnaire, 1 point was associated with each correct answer; thus, the level of food sustainability knowledge was rated on a score from 0 to 23 points.

### 2.3. Validation of the Food Sustainability Knowledge Questionnaire

The questions were defined based on a questionnaire already reported in the literature [24], adapted according to the concepts outlined in the Food and Agriculture Organization of the United Nations (FAO) report on Sustainable Diets [5] and Biodiversity and the Double Pyramid 2016 drawn up by the Barilla Foundation [21], after consultation with experts.

Initially, the questionnaire included 22 items and was tested with a pilot sample of 72 students to perform preliminary validation analyses, such as analyses of the item difficulty and item discrimination index. Based on these results (data not shown), the most critical questions were removed or revised. Afterward, a validation of the questionnaire was performed using the data collected from the control group, for which the sample size was previously determined according to the “rule of thumb” (*n*:p) [25]; the results are provided in Supplementary Materials S1. Responses from the 1st—baseline—data collection (T0) were used to carry out item analysis (Table S1). Each question was evaluated using the item difficulty index (percentage of correct responses for each single item) and item discrimination index (point-biserial correlation between the single-item score and the total score). Most of the questions had a difficulty index between 0.2 and 0.8, whereas for only three items, the percentage of correct answers was above 80% (two in the first and one in the second section), but none were properly answered by the entire sample and none of the questions had a difficulty index lower than 0.2. A slightly low correlation (item discrimination index of <0.2) between the total score and single items was found for three items in the “Environmental impact of foods” section, while one item, in the same section, showed a poor correlation index (0.010). Internal consistency was tested for both constructs separately and for the total questionnaire by running Cronbach’s alpha reliability test. The overall questionnaire internal consistency was acceptable (Cronbach’s α = 0.656) (Table S2). Also, Spearman’s correlation was applied to measure the temporal stability (test–retest reliability), considering the total score and the score of the two sections obtained at both data collection points (T0 and T1). A significant correlation was found between T0 and T1 (*p* < 0.001). An acceptable level of correlation was observed between total scores (ρ = 0.475, *p* < 0.001) and individual answers (ρ = 0.426, *p* < 0.001) (Table S3). In addition, a medium to high percentage of identical responses to the same item was found (62% for the total questionnaire).

Lastly, construct validity was examined by applying a nonparametric Mann–Whitney U test for independent samples, investigating possible differences in scores comparing students who were not educated on food sustainability issues (T1—control group) and students who received food sustainability lessons as part of the intervention (T1—intervention group). Knowledge was significantly higher (*p* < 0.001) in the intervention group; therefore, the questionnaire’s validity was confirmed (Table S4).

### 2.4. Statistical Analysis

The Kolmogorov–Smirnov test was performed to check the normality of the data distribution. According to the results, continuous variables are reported as the mean and standard deviation (SD) or median and interquartile range (IQR), while categorical variables are expressed as frequencies and percentages. In both the control and intervention groups, subjects with low, medium, or high nutrition or food sustainability knowledge were distinguished by subdividing them based on the tertiles of the knowledge scores at baseline, and the Chi-squared test was performed to assess changes in the distribution among tertiles between the two periods. A descriptive analysis of the number of correct answers is also reported. Within- and between-group comparison analysis for continuous variables was performed by respectively applying the Wilcoxon non-parametric test for paired samples and the Mann–Whitney non-parametric test for independent samples. Finally, an assessment of canteen users’ satisfaction was carried out through descriptive analysis, and the results are presented as numbers and percentages. All statistical analyses were carried out by IBM SPSS Statistics for Macintosh, version 28.0 (Armonk, NY, USA: IBM Corp.), taking *p* < 0.05 as the significance threshold.

## 3. Results

### 3.1. Subjects’ Characteristics

A total of 264 and 252 apprentice chefs were enrolled in the control and intervention groups, respectively. In both groups, the majority were male (control 69%, intervention 81%), and subjects in the two samples were comparable in terms of age (median, IQR, control: 22, 19–25; intervention: 21, 19–24). Regarding educational background, the share of students with hospitality training was significantly higher in the intervention group (58% vs. 28%, *p* < 0.001).

### 3.2. Nutrition and Food Sustainability Knowledge

The knowledge scores of the control and intervention groups are shown for both data collections (T0 and T1) in Table 1. Considering the total score, the level of nutrition knowledge significantly improved over time in both the control (*p* = 0.033) and intervention (*p* < 0.001) groups. Looking at individual constructs, the increase in nutritional knowledge was significant across all sections for the intervention group (*p* < 0.001), but not for the control group. On the contrary, a higher food sustainability knowledge was found only in the intervention group when prospectively compared (*p* < 0.001), considering both single constructs and the total score. The between-group comparison showed a higher median score in the intervention group (*p* < 0.001) at the end of the intervention for both the outcome variables, but the nutrition knowledge score was higher in the intervention group than in the control group, even at the beginning of the intervention (*p* < 0.001). Contrarily, the students’ food sustainability knowledge was comparable at baseline.

In addition, Table 2 shows the percentages of correct answers obtained at baseline and T1 in both groups, showing a good level of nutrition knowledge in both groups at baseline, mainly regarding the nutrient content of foods and the connection between diet and health status. On the other hand, food sustainability knowledge was lower in all students, regardless of the group.

For both nutrition and food sustainability knowledge, the distribution among the tertiles changed significantly between the two periods (*p* < 0.001) in subjects undergoing the intervention. The percentage having a high level of nutrition knowledge (score > 67) greatly increased over time (from 46% to 76%). At the same time, the number of subjects with a low level of knowledge (score ≤ 60) decreased (from 35% to 9%). The same pattern was found in relation to learning about the environmental impacts of food and diet, with an increase in the share of apprentice chefs having a high level of knowledge (score > 13) (from 35% to 77%) and a decrease in those reporting low knowledge scores (score ≤ 11) (from 38% to 12%). On the contrary, the control group remained largely stable, and no significant differences were found.

### 3.3. Acceptability of the Menus

Out of 58 menus, 57 received teacher approval and were served in the school canteen. The acceptance of almost all of the proposed menus reflects a conservative approach adopted by the school’s teachers, who worked with the students to find a solution even for menus that presented some issues. Turning to the acceptability of menus by diners, the subjects who replied were mostly male (58%), from 18 to 29 years old (74%), and mostly students (73%). Overall, meat menus were the most selected (37%), followed by plant-based (30%) and fish (17%) offers. The majority of users (80%) enjoyed the chosen menus and declared no leftovers (84%). Only 10% rated the portion sizes as abundant or excessive, while two-thirds and one-quarter of users considered them as adequate or meager, respectively. Lastly, the level of menu appreciation was medium–high (≥3) for 88% of the cafeteria customers, and 83% enjoyed the educational project and the option to pre-order the meal. The results of the satisfaction questionnaire are reported in Table S5 (Supplementary Materials S2).

## 4. Discussion

The present study assessed the effectiveness of an educational intervention aimed at increasing future chefs’ awareness and knowledge of the close connection between diet, health, and environment, as well as providing them with proper skills enabling them to prepare nutritionally balanced and eco-friendly menus.

The level of nutrition knowledge was medium–high in both groups at baseline, and it had increased at the end of the educational program in the intervention group, proving the efficacy of the educational intervention. However, an increase in nutrition knowledge was also registered for the control group. This may be warranted by the setting, even though no direct intervention was provided to these students. Indeed, engagement in school life, sharing with schoolmates, and attending the indoor cafeteria may have exposed them to new stimuli and experiences leading to an increase in their knowledge. This hypothesis is supported by the Social–Ecological Model theory according to which the social–environmental sphere is a strong determinant of behavioral changes in individuals [26], including the role of peers in enhancing knowledge about food [27]. The Social–Ecological Model has also been reported in the Ottawa Charter for Health Promotion [28] and advocates how individuals and the environment are extremely connected, representing the conceptual basis of public health “setting approach” interventions [29,30]. The lower nutrition knowledge of the control group at baseline could be related to their lower training in food science and the different admission requirements for the ALMA courses involved in the educational project. In fact, in contrast to the Cooking and Basic Pastry Techniques courses chosen as the control group [31,32], the Advanced Course in Italian Cuisine, chosen as the intervention group, represents a professionalizing course that requires basic knowledge of cooking techniques, defined according to the students’ educational background. Only students who have graduated from the Hospitality Academy or attended an equivalent cooking course, or with at least 2 years of kitchen experience, are eligible to enroll in the course [15]. For food sustainability, poor knowledge was observed in both groups, even though it increased in the intervention group. This finding probably reflects the current culinary programs mostly integrating nutritional components [33,34] without including other aspects of sustainability, which have only recently become a subject of interest to be incorporated into dietary recommendations [35,36] and chef practice [16,37,38]. In addition, notions should be taught to culinary students not only through lectures but, more importantly, by integrating them into culinary techniques to be applied in their future careers [39]. On this point, the integration of the teaching program with the preparation of nutritionally adequate and environmentally sustainable menus is a notable strength of this project.

Regarding nutrition knowledge, the total score obtained by the apprentice chefs was in line with the results reported for the sample of university students with a nutrition background enrolled by Rosi et al. [23] to validate the questionnaire applied in this study. Considering the Italian adult population, Aureli and colleagues recently validated a 12-item questionnaire [40], but in this example, the instrument aimed at investigating the attitudes of individuals, without specifically investigating their actual knowledge.

Some studies on the topic of nutrition and food sustainability knowledge are reported in the literature; however, comparisons are difficult due to the peculiarities of the study design and target population of our study. A recent study underlined a poor knowledge of nutritional guidelines among young Indonesian chefs [41], who instead expressed a greater interest in food techniques, including preparation, processing, presentation, and plating, to make their dishes more attractive. Two studies explored the level of knowledge, perceptions, and expectations about food sustainability in higher education, specifically in Spanish [42] and Georgian [43] university communities. The questionnaires applied in these studies differ, however, from the one we used as they addressed a different target population; there are also differences in the number of items and type of questions, which referred more to attitudes toward food sustainability, rather than assessing knowledge. At the same time, some instruments have been validated on culinary students, but they aimed at assessing beliefs and attitudes toward food, and cooking skills, rather than nutrition and food sustainability knowledge [13,44,45,46]. Only a recent study [12] on U.S. culinary students addressed the issue of food sustainability, delving into their attitudes and beliefs about the role of chefs in encouraging healthy diets and sustainable food systems. The students interviewed listed price, taste, and convenience as priorities when choosing food. However, they recognized the importance of encouraging healthy and sustainable food choices through food services.

To the best of our knowledge, this is the first study assessing food sustainability knowledge in a sample of apprentice chefs. Despite the novelty and importance of this study, some limitations should be pointed out. To begin with, randomized allocation of subjects between the control and intervention groups was not possible. This led to selection bias, particularly concerning the educational background of the participants, which resulted in non-comparability of the two samples at baseline in terms of nutrition knowledge. In addition, the food sustainability questionnaire developed in our study performed well in distinguishing students who were expected to have a higher knowledge from those who did not have a food sustainability background, but suboptimal values of Cronbach’s alpha and correlation over time were found. Considering the low discrimination indices of some items in the second section, a revision of these questions would be appropriate to strengthen the reliability of this tool. Finally, this study was designed to represent the local reality of the school. Although ALMA is recognized worldwide as one of the most important training centers for the Italian catering and hospitality industries, future national and international implementations will increase the reliability of these promising results.

## 5. Conclusions

In conclusion, the present study was the first concrete example of incorporating nutritional and environmental sustainability concepts into the practices of ALMA culinary students. The educational intervention proved to be effective in improving ALMA students’ knowledge about nutrition and the environmental impact of food production and consumption, confirming the importance of promoting education in the related fields to meet a central public health need in supporting the dietary behavioral shift towards healthy and sustainable dietary choices. However, as chefs have increasingly gained visibility, often assuming the role of a health promoter, especially in social media communities and television programs, it is important to emphasize that their action in this nutrition transition should be constantly supported by experts in the field and health professionals.

Finally, the validation analysis of the food sustainability knowledge questionnaire pointed out some weaknesses that should be treated as a guide for future developments. In light of this, our results serve as a starting point from which a reliable tool can be developed to assess chefs’ knowledge of the environmental impact of food, especially considering the growing number of educational initiatives targeted at food service professionals and the need to evaluate and monitor their effectiveness. 

## Figures and Tables

**Table 1:** Within- and between-group comparisons of knowledge scores (median IQR) obtained at T0 and T1.

	T0			T1			*p*-Value ^§^	
Constructs	Control *n* = 264	Intervention *n* = 252	*p*-Value *	Control *n* = 264	Intervention *n* = 252	*p*-Value *	Control T0–T1	Intervention T0–T1
NK								
NK 1 (0–6)	4 (3–5)	4 (3–5)	0.008	4 (3–5)	5 (5–6)	<0.001	0.002	<0.001
NK 2 (0–49)	35 (31–38)	37 (34–40)	<0.001	35 (32–38)	38 (36–41)	<0.001	0.301	<0.001
NK 3 (0–10)	7 (6–8)	7 (6–8)	0.428	7 (6–8)	8 (7–9)	<0.001	0.326	<0.001
NK 4 (0–19)	15 (12–16)	15 (13–17)	0.055	15 (13–17)	17 (15–18)	<0.001	0.017	<0.001
NK 5 (0–6)	4 (3–4)	4 (3–4)	0.930	4 (3–4)	4 (4–5)	<0.001	0.781	<0.001
Total (0–90)	64 (57–69)	67 (60–71)	<0.001	64 (59–69)	73 (68–76)	<0.001	0.033	<0.001
FSK								
FSK 1 (0–9)	5 (4–6)	5 (4–6)	0.456	5 (4–6)	7 (6–8)	<0.001	0.783	<0.001
FSK 2 (0–14)	7 (6–8)	7 (6–8)	0.354	7 (6–8)	9 (8–10)	<0.001	0.221	<0.001
Total (0–23)	12 (10–14)	13 (11–14)	0.397	13 (11–14)	16 (14–17)	<0.001	0.258	<0.001

* Non-parametric Mann–Whitney U test for independent samples for between-group analyses within each period. ^§^ Non-parametric Wilcoxon test for paired samples between periods within the control and intervention groups separately. Possible scores for each questionnaire section are shown within parentheses in the first column. T0: 1st—baseline—data collection; T1: 2nd—final—data collection; NK: nutrition knowledge; FSK: food sustainability knowledge; NK 1: experts’ recommendation; NK 2: nutritional content of food; NK 3: health aspects of food and diet; NK 4: relationship between diet and diseases; NK 5: proper food choices, FSK 1: basic notions about food sustainability; FSK 2: environmental impact of food.

**Table 2:** Descriptive analysis of correct answers obtained at T0 and T1 in both groups.

	T0		T1	
Constructs	Control *n* = 264	Intervention *n* = 252	Control *n* = 264	Intervention *n* = 252
	Correct answers (%)	Correct answers (%)	Correct answers (%)	Correct answers (%)
NK				
NK 1 (0–6)	63 (19)	67 (21)	66 (19)	87 (10)
NK 2 (0–49)	70 (24)	75 (23)	71 (24)	77 (22)
NK 3 (0–10)	72 (25)	71 (27)	71 (25)	78 (19)
NK 4 (0–19)	74 (12)	76 (11)	76 (12)	86 (10)
NK 5 (0–6)	59 (22)	60 (22)	59 (23)	73 (21)
Total (0–90)	70 (22)	73 (21)	71 (22)	80 (19)
FSK				
FSK 1 (0–9)	57 (24)	59 (20)	57 (20)	74 (15)
FSK 2 (0–14)	50 (22)	52 (22)	52 (22)	63 (22)
Total (0–23)	53 (23)	54 (21)	54 (21)	67 (20)

Results are reported as the average percentage (SD) for each construct of both questionnaires. T0: 1st—baseline—data collection; T1: 2nd data collection; NK: nutrition knowledge; FSK: food sustainability knowledge; NK 1: experts’ recommendation; NK 2: nutritional content of food; NK 3: health aspects of food and diet; NK 4: relationship between diet and diseases; NK 5: proper food choices, FSK 1: basic notions about food sustainability; FSK 2: environmental impact of food.

## Data Availability

The data presented in this study are available upon reasonable request from the corresponding author (A.R.).

## Supplementary Materials

The following supporting information can be downloaded at: https://www.mdpi.com/article/10.3390/nu16040537/s1, Supplementary Materials S1: Validation of a food sustainability knowledge questionnaire; Supplementary Materials S2: Results of satisfaction questionnaire. Table S1. Item analysis for the 23 questions of the food sustainability knowledge questionnaire; Table S2. Internal consistency; Table S3. Temporal stability (test-retest reliability); Table S4. Construct validity; Table S5. Results of satisfaction questionnaire.
