# Supplementary material for: Promoting Nutrition and Food Sustainability Knowledge in Apprentice Chefs: An Intervention Study at The School of Italian Culinary Arts—ALMA
Source: Nutrients. 2024 Feb 15;16(4):537. doi: 10.3390/nu16040537 (PMC10892208; doi:10.3390/nu16040537)
Supplement: Supplementary file 1 [file nutrients-16-00537-s001.zip › Supplementary_material_S1.pdf]

Supplementary Material S1

Table S1 Item analysis for the 23 questions of the food sustainability knowledge questionnaire.

Item	Item Difficulty (% Correct Answers)	Item Discrimination (*r* Value)
Construct 1: Basic notions about food sustainability		
1 – sustainable diet definition	79%	0.260
2 – animal-based foods	81%	0.250
3 – imported goods	50%	0.275
4 – red meat	68%	0.340
5 – seasonal and low-processed products	95%	0.309
6 – carbon footprint definition	32%	0.362
7 – water footprint definition	48%	0.368
8 – ecological footprint definition	34%	0.279
9 – Double Pyramid Model concept	32%	0.418
Construct 2: Environmental impact of foods		
10 – impact of milk	43%	0.010
11 – impact of fish	28%	0.157
12 – impact of pork	80%	0.314
13 – impact of pasta	64%	0.330
14 – impact of hard cheese	36%	0.254
15 – high impact foods	25%	0.381
16 – low impact foods	48%	0.329
17 – transportation and impact of fruit and vegetables	60%	0.266
18 – breeding and impact of meat	72%	0.323
19 – farming and cooking, and impact of cereals	28%	0.178
20 – impact of local products	22%	0.186
21 – eco-friendly diet and human health	75%	0.296
22 – food waste reduction	39%	0.314
23 – Mediterranean Diet as a sustainable model	86%	0.284

Items are reported divided into the two constructs.

Table S2 Internal consistency.

Section	Score range	Baseline FSK Score	Cronbach's Alpha
		Median (IQR)	
1 – Basic notions about food sustainability	0-9	5.0 (4.0-6.0)	0.574
2 – Environmental impact of foods	0-14	7.0 (6.0-8.0)	0.490
Total questionnaire	0-23	12.0 (10.0-14.0)	0.656

FSK: food sustainability knowledge.

Table S3 Temporal stability (test-retest reliability).

Section	Baseline SK Score (T0)	Follow-up SK Score (T1)	Correlation (Total Scores)		Correlation (Individual answers)	
	Median (IQR)	Median (IQR)	*rho*	*p*-Value*	*rho*	*p*-Value*
1 – Basic notions about food sustainability	5.0 (4.0-6.0)	5.0 (4.0-6.0)	0.347	<0.001	0.407	<0.001
2 – Environmental impact of foods	7.0 (6.0-8.0)	7.0 (6.0-8.0)	0.373	<0.001	0.434	<0.001
Total questionnaire	12.0 (10.0-14.0)	13.0 (10.3-14.0)	0.475	<0.001	0.426	<0.001

*Spearman's correlation test with significance of $p < 0.05$
FSK: food sustainability knowledge.

Table S4 Construct validity.

Section	FSK score Group 1 Median (IQR)	FSK score Group 2 Median (IQR)	*p*-Value*
1 – Basic notions about food sustainability	5.0 (4.0-6.0)	7.0 (6.0-8.0)	<0.001
2 – Environmental impact of foods	7.0 (6.0-8.0)	9.0 (8.0-10.0)	<0.001
Total questionnaire	13.0 (10.3-14.0)	16.0 (14.0-17.0)	<0.001

* Test U di Mann-Whitney
FSK: food sustainability knowledge
Group 1: students enrolled in Cooking Techniques and Basic Pastry Techniques courses in which lesson on food sustainability were not provided
Group 2: students enrolled in Advanced Course in Italian Cuisine in which lesson on food sustainability were provided
