# Supplementary material for: Promoting Nutrition and Food Sustainability Knowledge in Apprentice Chefs: An Intervention Study at The School of Italian Culinary Arts—ALMA
Source: Nutrients. 2024 Feb 15;16(4):537. doi: 10.3390/nu16040537 (PMC10892208; doi:10.3390/nu16040537)
Supplement: Supplementary file 1 [file nutrients-16-00537-s001.zip › Supplementary_material_S2.pdf]

Supplementary Material S2

Table S5 Results of satisfaction questionnaire.

**Information**	**Replies (*n*=3599)**
Gender	
Females	1528 (42)
Males	2071 (58)
User	
Student	2602 (73)
Teacher	947 (26)
Guess	50 (1)
Age group	
18-29	2681 (74)
30-40	611 (17)
40-50	282 (8)
> 50	25 (1)
Selected menu	
Meat	1327 (37)
Fish	1190 (33)
Vegetarian (or vegan)	1082 (30)
Would you have chosen a different menu?	
Yes	703 (20)
No	2896 (80)
Did you finish the entire meal?	
Yes	3029 (84)
No	570 (16)
How do you consider the size of the portions served?	
Insufficient	204 (5)
Meager	703 (20)
Adequate	2345 (65)
Abundant	321 (9)
Excessive	26 (1)
Express your satisfaction from 1 to 5	
1	125 (4)
2	280 (8)
3	831 (23)
4	1231 (34)
5	1132 (31)
Would you like to see this proposal, which includes online mode of choice and nutritional information, replicated?	
Yes	2998 (83)
No	601 (17)

Data are reported as absolute number (percentage).
